# *In vivo* Ultrasound and Photoacoustic Monitoring of Mesenchymal Stem Cells Labeled with Gold Nanotracers

**DOI:** 10.1371/journal.pone.0037267

**Published:** 2012-05-16

**Authors:** Seung Yun Nam, Laura M. Ricles, Laura J. Suggs, Stanislav Y. Emelianov

**Affiliations:** 1 Department of Biomedical Engineering, University of Texas at Austin, Austin, Texas, United States of America; 2 Department of Electrical and Computer Engineering, University of Texas at Austin, Austin, Texas, United States of America; University of Medicine and Dentistry of New Jersey, United States of America

## Abstract

Longitudinal monitoring of cells is required in order to understand the role of delivered stem cells in therapeutic neovascularization. However, there is not an imaging technique that is capable of quantitative, longitudinal assessment of stem cell behaviors with high spatial resolution and sufficient penetration depth. In this study, *in vivo* and *in vitro* experiments were performed to demonstrate the efficacy of ultrasound-guided photoacoustic (US/PA) imaging to monitor mesenchymal stem cells (MSCs) labeled with gold nanotracers (Au NTs). The Au NT labeled MSCs, injected intramuscularly in the lower limb of the Lewis rat, were detected and spatially resolved. Furthermore, our quantitative *in vitro* cell studies indicate that US/PA imaging is capable of high detection sensitivity (1×10^4^ cells/mL) of the Au NT labeled MSCs. Finally, Au NT labeled MSCs captured in the PEGylated fibrin gel system were imaged *in vivo*, as well as *in vitro*, over a one week time period, suggesting that longitudinal cell tracking using US/PA imaging is possible. Overall, Au NT labeling of MSCs and US/PA imaging can be an alternative approach in stem cell imaging capable of noninvasive, sensitive, quantitative, longitudinal assessment of stem cell behaviors with high spatial and temporal resolutions at sufficient depths.

## Introduction

Ischemic heart disease is one of the most common killers of American men and women, accounting for about half a million deaths every year [1], [2]. Recent experimental and preclinical research suggests that stem cell therapy can be a safe and clinically feasible alternative treatment for ischemic heart disease because stem cells can differentiate into multiple cell types, including vascular cells contributing to myocardial regeneration [3], [4]. It was previously demonstrated that tubulogenesis and differentiation of mesenchymal stem cells (MSCs) to vascular cell phenotypes can be enhanced within tissue-engineered matrices, indicating the possibility of stem cell delivery to ischemic regions to promote neovascularization [5]–[8]. In order to obtain a better understanding of the role of stem cells in neovascularization, continuous monitoring of the distribution of stem cells, as well as interaction of the stem cells with their microenvironment, is essential. Therefore, there is a need for a stem cell imaging technique that is not only noninvasive and sensitive, but also capable of quantitative, longitudinal assessment of stem cell behaviors with high spatial resolution.

Unfortunately, none of the current stem cell imaging modalities can satisfy all requirements simultaneously. Magnetic resonance imaging (MRI), with contrast agents based on superparamagnetic iron oxide, has been widely researched for stem cell monitoring. Although MRI can achieve relatively high resolution and long-term cell tracking, it has low sensitivity with the current contrast agents [9]–[12]. Radioactive imaging, including single photon emission computed tomography (SPECT) and positron emission tomography (PET), has a fair sensitivity with direct labeling of stem cells. However, high spatial resolution and long-term imaging are not obtainable with SPECT and PET due to intrinsic limitations, such as the short half-lives of radioisotopes [9]–[12]. While direct cell labeling with MRI and radioactive imaging cannot provide information about cell proliferation, reporter gene labeling is capable of tracking cell division with radioactive and optical imaging techniques. Yet, imaging with reporter gene labeling is still limited for clinical approaches due to cell safety issues, such as immunogenic responses and transfection [9]–[12]. Optical imaging modalities, including fluorescence and bioluminescence imaging, have the highest sensitivity and highest resolution among the available imaging methods, but they are restricted by shallow penetration depth and phototoxicity, thus limiting the clinical applicability [9], [13].

In this study, we explored the feasibility of imaging stem cells using ultrasound-guided photoacoustic (US/PA) imaging [14]–[18]. In photoacoustic imaging, a photoacoustic wave is generated by thermal expansion of tissue after absorption of a short laser pulse. The magnitude of the photoacoustic wave is proportional to the laser fluence and to the optical absorption coefficient. Similar to ultrasound, photoacoustic imaging provides a penetration depth of several centimeters and sub-millimeter spatial resolution [19]–[21]. In addition, similarities between photoacoustic and ultrasound imaging systems enable the hybrid implementation of the two modalities, thus providing synergetic benefits, such as the capability of visualizing morphological, functional, and molecular properties [22]. Thus, the US/PA imaging method may have great potential for continuous *in vivo* monitoring of MSC behavior and neovascularization promoted by MSCs [23], [24].

**Figure 1:**
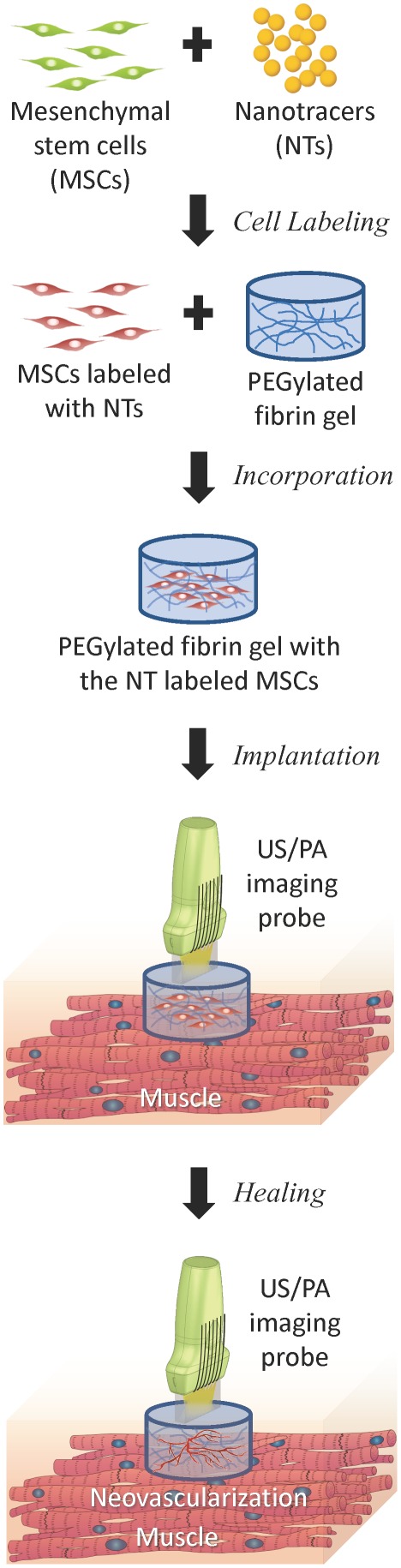
Diagram of the procedure for monitoring mesenchymal stem cells (MSCs) *in vivo*. Once MSCs are loaded with nanotracers, the labeled MSCs are entrapped in the PEGylated fibrin gels and implanted at the ischemic region. The PEGylated fibrin gels promote MSC differentiation toward a vascular cell type, thus contributing to regeneration. Both MSC distribution and neovascularization can be monitored using the combined ultrasound and photoacoustic imaging of cells loaded with nanotracer such as gold plasmonic nanoparticles.

Unfortunately, MSCs do not have sufficient optical absorption and cannot be directly visualized using US/PA imaging. However, as depicted in Figure 1, the optical properties of MSCs can be altered by labeling MSCs with gold nanotracers (Au NTs) and using US/PA imaging to monitor and track the cells. Therefore, in this study, *in vivo* as well as *in vitro* experiments were performed to evaluate *in vivo* detectability, sensitivity, quantification, and long-term MSC tracking ability with the suggested method. To demonstrate the feasibility of noninvasive monitoring of MSCs labeled with Au NTs, *in vivo* US/PA imaging of the nanotracer labeled MSCs injected intramuscularly in the lower limb of the rat was conducted. Following *in vivo* studies, sensitivity as well as quantification of the ultrasound and photoacoustic imaging method were assessed using a tissue mimicking gelatin phantom with inclusions of Au NT labeled and non-labeled MSCs. Furthermore, Au NT labeled MSCs cultured *in vitro* in a PEGylated fibrin gel system were imaged over a one week time period to verify the possibility of longitudinal cell tracking using photoacoustic imaging. Finally, longitudinal *in vivo* monitoring of spatial distribution of the PEGylated fibrin gel containing the Au NT labeled MSCs was demonstrated at the three different time points (day 3, 7, and 10 after the injection).

## Results

### Au NT labeling of MSCs

**Figure 2:**
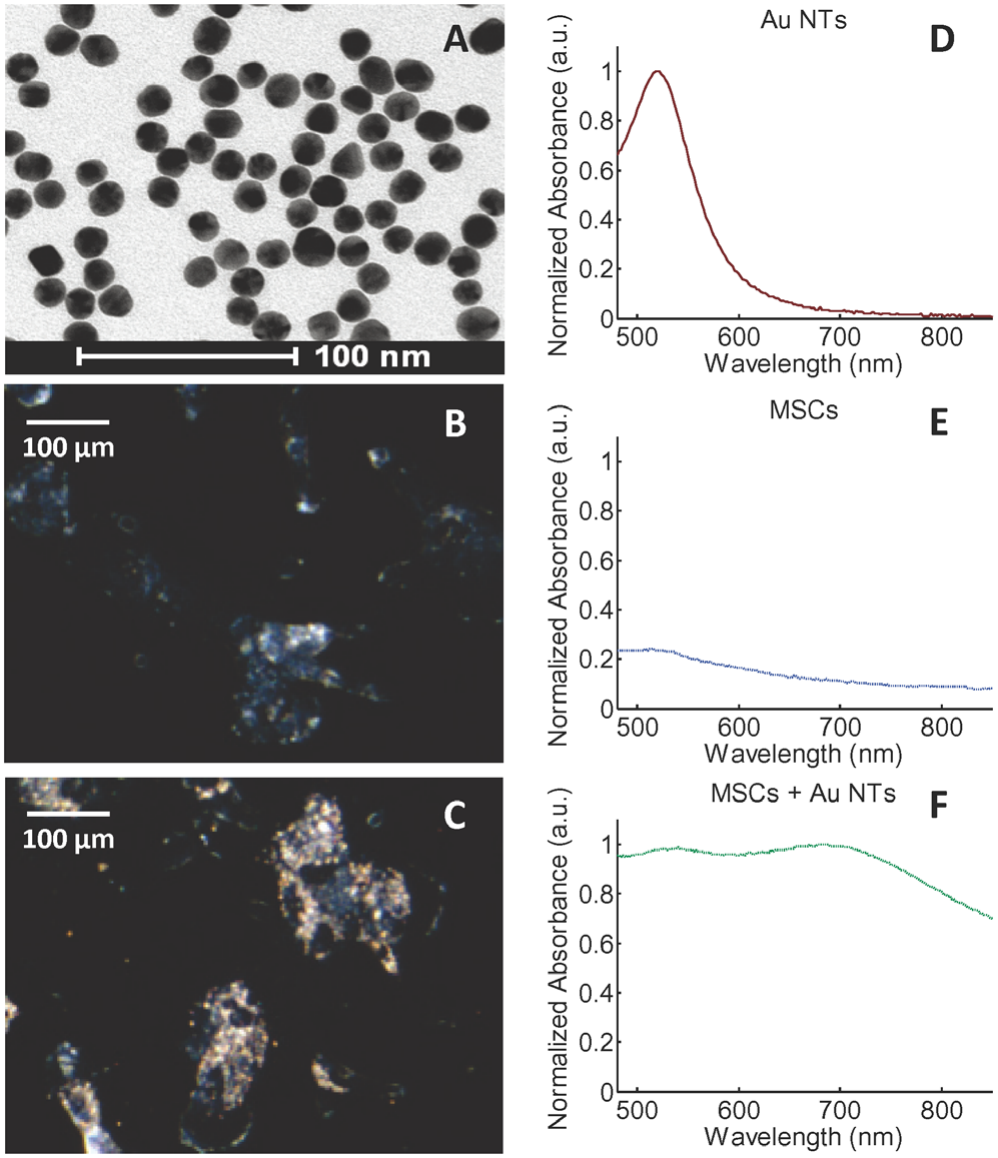
Gold nanotracer (Au NT) labeling of MSCs. (A) The TEM image of 20 nm gold nanotracers and the dark field images (20× magnification) of MSCs without and with nanotracer loading (B and C, respectively). The normalized absorbance spectra of Au NTs, MSCs and the Au NT labeled MSCs (D,E, and F, respectively).

Gold nanoparticles are one of the most widely used nanotracers for imaging cells of various types due to their excellent biocompatibility, as well as tunable optical properties [25]–[27]. It was recently demonstrated that gold nanoparticles can be safe and effective nanotracers for longitudinal tracking of MSCs [28]. Specifically, cell viability, cell proliferation, and cell differentiation were not significantly affected by nanotracer uptake of various sizes and surface coatings [28]. For this study, MSCs were labeled with 20 nm Au NTs (Figure 2A), which were prepared and used as previously described [28]. MSCs were incubated with cell culture medium including Au NTs for 24 hours while control cells were not. After MSC culture with nanotracer solutions, darkfield images (Figure 2B and Figure 2C) were obtained to assess nanotracer uptake. While the non-labeled MSCs in Figure 2B appear bluish, Figure 2C shows orange colored nanotracers which endocytosed in the MSCs. Furthermore, as shown in Figure 2D and Figure 2F, passive loading of Au NTs to MSCs resulted in a red-shift and peak broadening in their absorbance spectrum due to nanoparticle aggregation inside the cells [28], [29]. In addition, inductively coupled plasma mass spectrometry (ICP-MS) was used to quantify nanotracer loading in the cells. It was found that the average nanotracer loading was [4.53±0.04]×10^5^ nanotracers per MSC.

### In vivo monitoring of Au NT labeled MSCs using ultrasound and photoacoustic imaging

**Figure 3:**
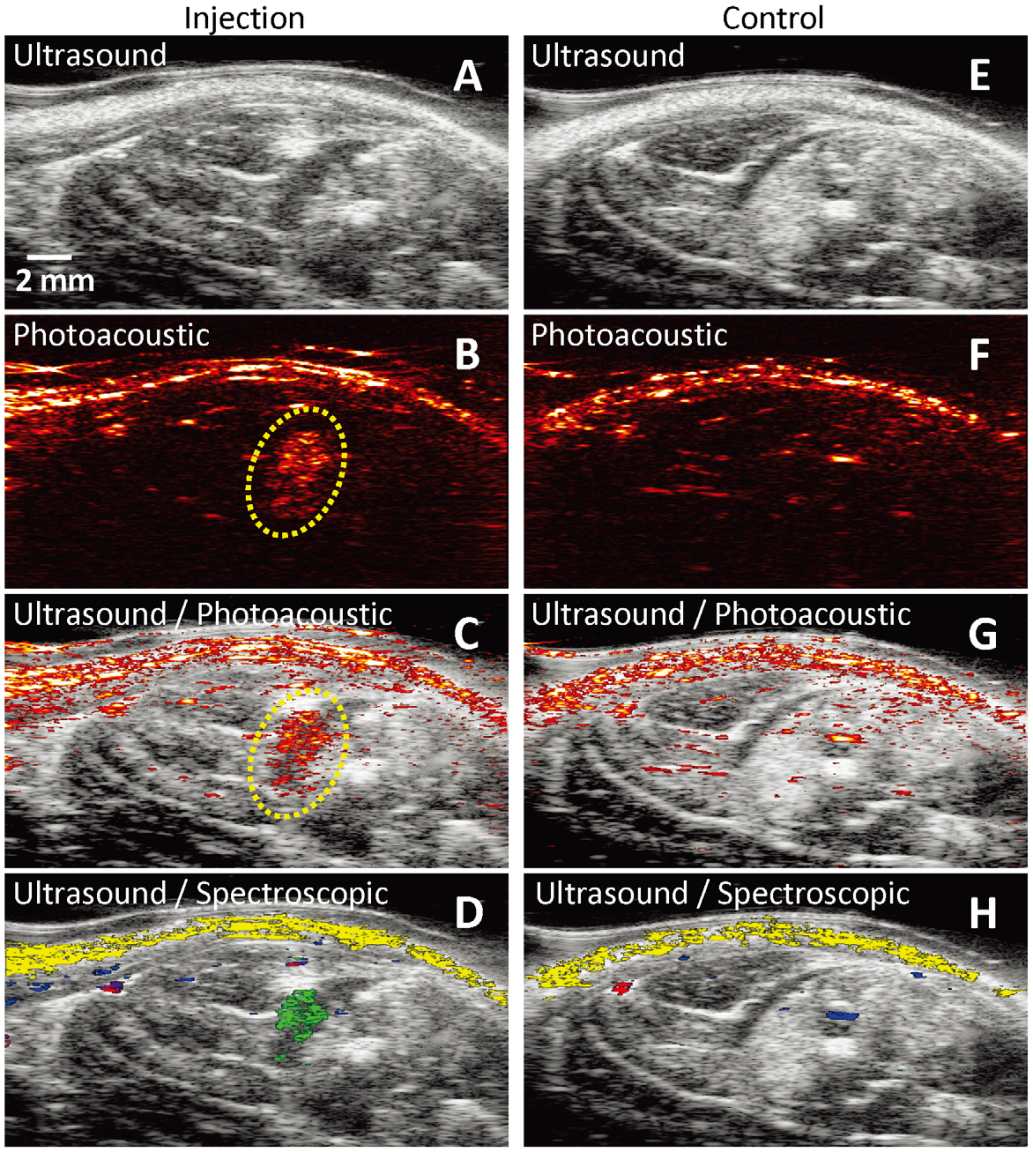
*In vivo* monitoring of Au NT labeled MSCs using combined ultrasound and photoacoustic (US/PA) imaging. (A–D) *In vivo* ultrasound, photoacoustic, US/PA, and US/spectroscopic images of the LGAS in which PEGylated fibrin gel containing Au NT loaded MSCs (1×10^5^ cells/mL) was injected. PEGylated fibrin gel location is outlined with yellow dotted circle. Injection depth was about 5 mm under the skin. (E–H) Control at the region of the LGAS of the other hind limb without any injection. Photoacoustic images were acquired at the wavelength of 760 nm with a fluence of 11 mJ/cm^2^. Spectral (650–920 nm) analysis of photoacoustic signal was able to differentiate skin (shown in yellow), oxygenated (red) and deoxygenated (blue) blood, and Au NT loaded MSCs (green). The images measure 23 mm laterally and 12.5 mm axially.

The unique optical properties of Au NT labeled MSCs with a broad range of wavelengths, including in the near-infrared (NIR) optical window in biological tissue, allow noninvasive photoacoustic imaging as well as spectral analysis to distinguish MSCs from background tissue (Figure S1A). To demonstrate the feasibility of noninvasive *in vivo* monitoring of MSCs, the Au NT labeled MSCs were injected intramuscularly in the hind limb of the rat and visualized using US/PA imaging. A PEGylated fibrin gel with Au NT loaded MSCs (1×10^5^ cells/mL) was injected intramuscularly in the lateral gastrocnemius (LGAS) of an anesthetized Lewis rat and immediately imaged at a range of wavelengths from 650 nm to 920 nm with a fluence of 11 mJ/cm^2^, which was verified to be safe (Figure S2) and lower than the maximum allowable laser fluence [30]. In addition, a region of the LGAS of the other hind limb without any injection served as a control. Figures 3A, 3B, 3C, 3D represent ultrasound, photoacoustic, US/PA, and US/spectroscopic images, respectively, of the LGAS in which PEGylated fibrin with Au NT loaded MSCs was injected. The ultrasound image shows the structural information of the lower limb, but the location of MSCs cannot be identified. However, the photoacoustic and US/PA images clearly show the location of the nanotracer signal from MSCs in the gel outlined in yellow. In order to distinguish the photoacoustic signal from the Au NT labeled MSCs from surrounding tissue, spectral analysis can be performed, which augments photoacoustic imaging. Specifically, the unique absorbance of Au NT labeled MSCs compared with those of oxygenated and deoxygenated hemoglobin (Figure S1A) can be accurately detected using multiwavelength photoacoustic imaging (Figure S1B). Therefore, as shown in Figure 3D, the Au NT labeled MSCs (shown in green) can be clearly distinguished from other tissue constituents, such as oxygenated (red) hemoglobin, deoxygenated (blue) hemoglobin, and skin (yellow), by spectral analysis. As shown in Figures 3E, 3F, 3G, 3H of the control, photoacoustic signals were generated only from the background tissue (mainly skin and blood). This result indicates that US/PA imaging has the capability of monitoring MSCs labeled with nanotracers noninvasively.

### Quantification of Au NT labeled MSCs

**Figure 4:**
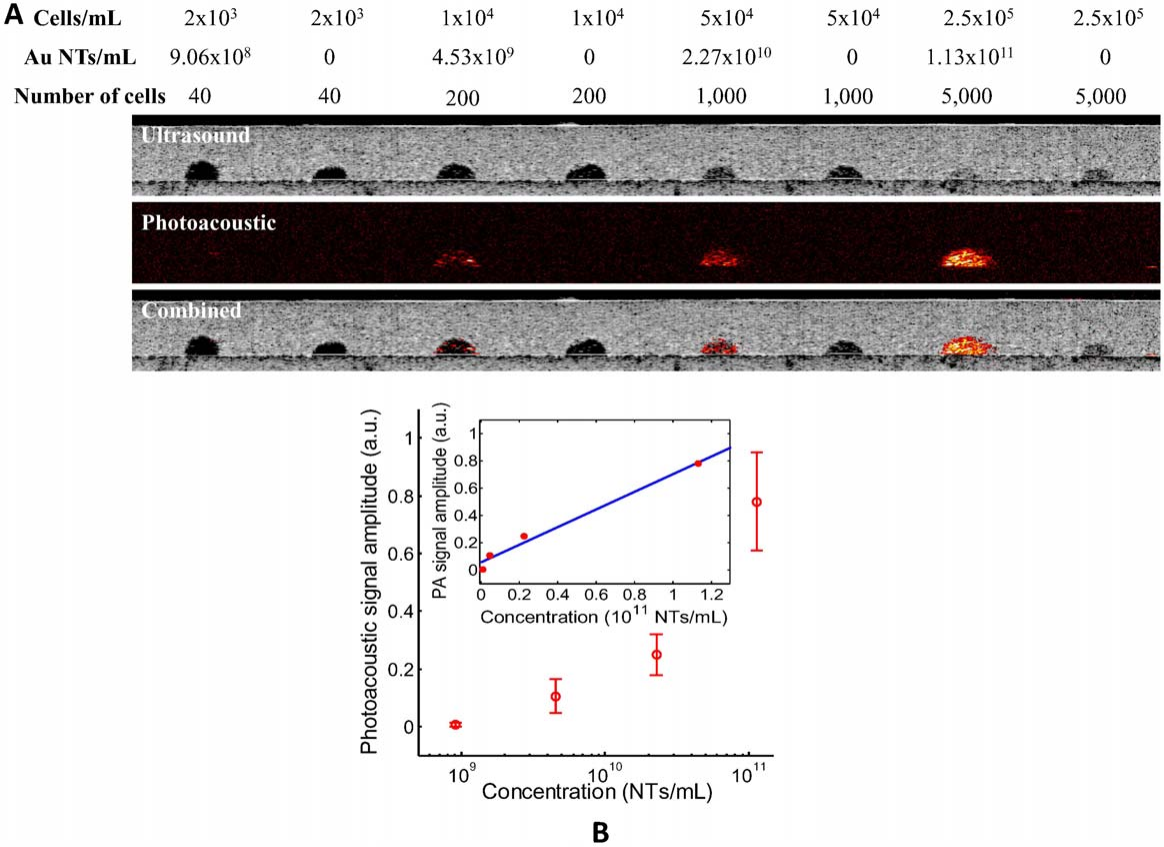
Quantification of Au NT labeled MSCs. (A) Ultrasound (top), photoacoustic (middle), and US/PA images (bottom) of the gelatin phantom with inclusions containing different concentrations of MSCs. Photoacoustic images were obtained at a wavelength of 750 nm with a fluence of 5.1 mJ/cm^2^. All images measure 98 mm laterally and 7.7 mm axially. (B) The quantitative analysis of photoacoustic signal. The mean and the standard deviation of the photoacoustic signal amplitude as a function of the nanoparticle concentration is shown in the outer graph in a semi-logarithmic scale. The inset graph presents the linear regression fit (with an R^2^ value of 0.984) of the mean values of the photoacoustic signal amplitude as a function of the nanoparticle concentration in a linear scale.

In addition to the *in vivo* detectability of the Au NT labeled stem cells, accurate quantification of stem cells with high sensitivity is one of the essential requirements for effective stem cell tracking methods. To evaluate the sensitivity and cell quantification ability of photoacoustic imaging, an *in vitro* experiment using a tissue mimicking gelatin phantom was performed. Figure 4A shows the ultrasound, photoacoustic, and US/PA images of the tissue mimicking phantom with inclusions containing Au NT labeled or non-labeled MSCs at a wavelength of 750 nm. Photoacoustic signals were not detected from any of the inclusions with the non-labeled MSCs, regardless of the concentration of MSCs, because the cells themselves do not absorb light. Furthermore, the quantitative analysis of the photoacoustic signal amplitudes measured from the Au NT labeled inclusions, using a laser fluence of 5.1 mJ/cm^2^, is shown in Figure 4B. The means and standard deviations of the photoacoustic signal amplitudes were calculated from twelve subareas (0.4 mm×0.77 mm) in the Au NT labeled MSC inclusions as shown in the outer graph of Figure 4B on a semi-logarithmic scale. The inset graph presents the linear regression fit (with an R^2^ value of 0.984) of the mean values of the photoacoustic signal amplitudes as a function of the nanotracer concentration on a linear scale. The results indicate that the amplitude of the photoacoustic signal is proportional to the concentration of optical absorbers, which are the Au NTs loaded inside MSCs. The results also indicate that with a fluence of 5.1 mJ/cm^2^, a photoacoustic signal can be detected for a nanotracer concentration as low as 4.53×10^9^ NTs/mL, which corresponds to 1×10^4^ cells/mL and 200 cells. Therefore, the photoacoustic imaging method can precisely quantify MSC concentrations with excellent sensitivity better than other noninvasive stem cell tracking methods [20], [31].

### Longitudinal in vitro photoacoustic imaging of MSCs labeled with Au NTs

**Figure 5:**
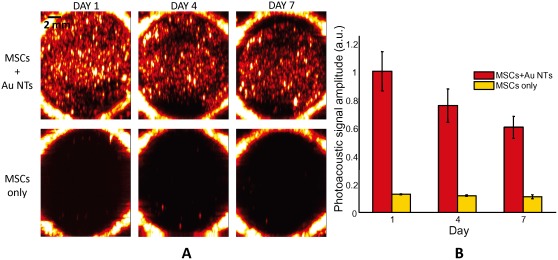
Longitudinal *in vitro* photoacoustic imaging of MSCs labeled with Au NTs. (A) Longitudinal photoacoustic images of MSCs loaded with Au NTs, or MSCs only, in a PEGylated fibrin gel at a wavelength of 750 nm with a fluence of 11 mJ/cm^2^. The MSCs in the PEGylated fibrin gel were cultured in a 24 well plate over a one week time period. While the MSCs without Au NT loading did not produce any photoacoustic signal, strong photoacoustic signals were detected from the MSCs loaded with Au NTs over a one week time period. The images measure 14.1 mm laterally and 16.6 mm axially. (B) The quantitative analysis of photoacoustic signal. The mean and the standard deviation of the photoacoustic signal amplitude from the PEGylated fibrin gels containing the MSCs with and without Au NT loading were shown in green and yellow colors, respectively.

Although we can achieve high sensitivity and cell quantification ability using the photoacoustic imaging, continuous cell tracking is essential to monitor MSC migration and proliferation. To demonstrate this, longitudinal (up to 7 days) photoacoustic images of MSCs loaded with Au NTs, or MSCs only, in PEGylated fibrin gels were acquired at a wavelength of 750 nm with a fluence of 11 mJ/cm^2^ (Figure 5A). The PEGylated fibrin gels were cast as previously described [5], [6], and MSCs were entrapped into the PEGylated fibrin gels at a concentration of 5×10^4^ cells/mL. The MSCs were cultured in the PEGylated fibrin gels in a 24 well plate over a one week period and imaged at three different time points (day 1, 4, and 7). As shown in Figure 5A, while the MSCs without Au NT loading did not produce any photoacoustic signal, strong photoacoustic signals were detected from the MSCs loaded with Au NTs over a one week time period. As we previously evaluated, nanoparticle loading decreased exponentially over time due to both cell division and exocytosis of the nanoparticles by the cells [28]. The photoacoustic signals from the PEGylated fibrin gels were quantitatively analyzed as shown in Figure 5B. Specifically, the means and standard deviations of the photoacoustic signal amplitudes obtained from nine subareas (2 mm×2 mm×2 mm) in the PEGylated fibrin gels containing the MSCs with and without Au NT loading were shown in red and orange colors in Figure 5B, respectively. Because the photoacoustic signal is proportional to the concentration of Au NTs as demonstrated above, the Au NT concentration at three different time points could be quantified to [2.27±0.31]×10^10^ NTs/mL, [1.71±0.27]×10^10^ NTs/mL, and [1.37±0.18]×10^10^ NTs/mL for day 1, 4, and 7, respectively. Furthermore, sensitivity of the photoacoustic imaging was enough to detect the labeled MSCs in spite of the decrease in Au NT loading. Hence, this result implies that photoacoustic imaging can be used to assess temporal behaviors of the Au NT labeled MSCs.

### Longitudinal in vivo monitoring of the spatial distribution of MSCs labeled with Au NTs using US/PS imaging

**Figure 6:**
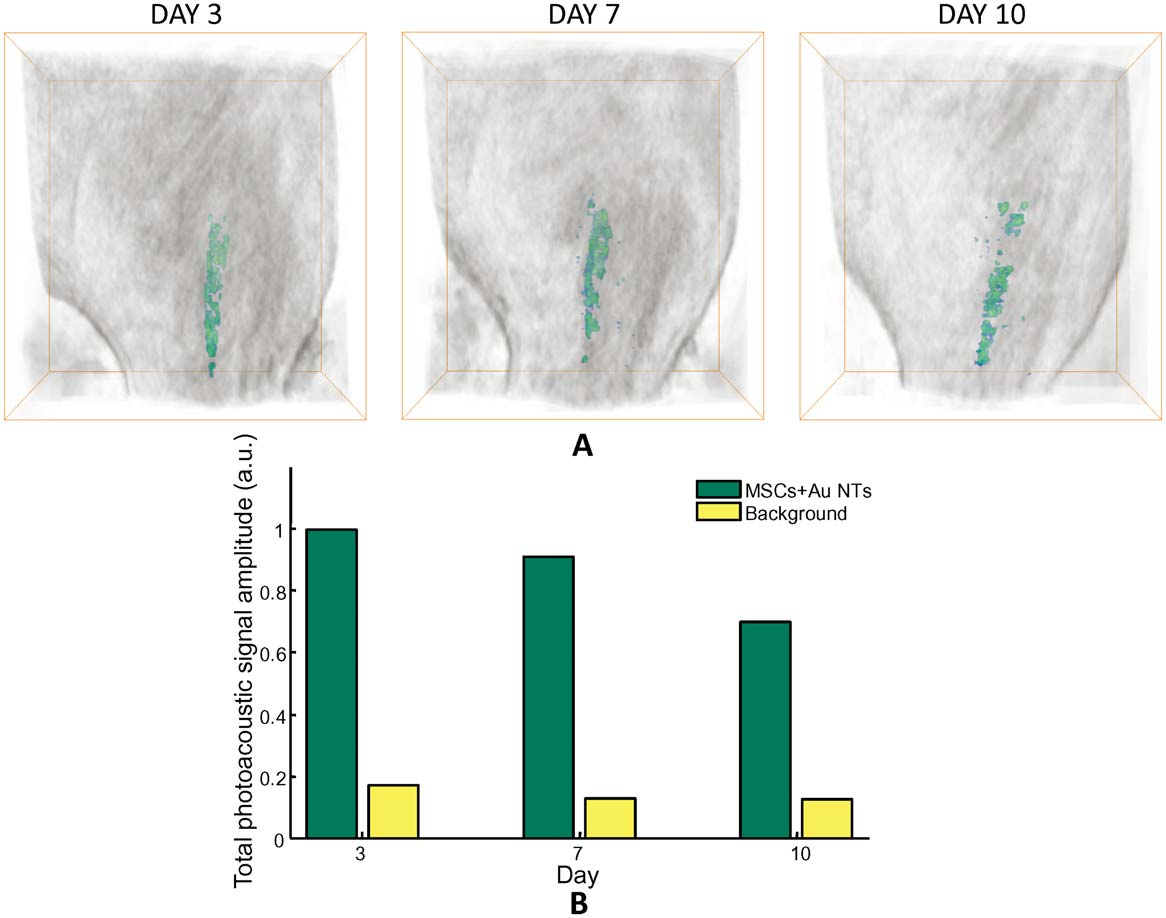
Longitudinal *in vivo* monitoring spatial distribution of MSCs labeled with Au NTs using US/PS imaging. (A) 3-D combined ultrasound and spectroscopic images of the rat hind limb in which the PEGylated fibrin gel containing Au NT labeled MSCs were injected (day 3,7, and 10). The MSCs labeled with Au NTs were distinguished using spectral analysis and presented in green color. The bounding box for each image measures 23 mm laterally, 12.5 mm axially, and 25 mm elevationally. (B) The quantitative analysis of photoacoustic signal. The photoacoustic signals at three different time points obtained from the Au NT labeled MSCs and the background tissue were summed and displayed in green and yellow colors, respectively.

Based on the longitudinal *in vitro* study, the Au NT labeled MSCs were longitudinally monitored *in vivo*. After injection of the PEGylated fibrin gel with Au NT loaded MSCs (5×10^4^ cells/mL, 300 µL) into the LGAS of the Lewis rat, the labeled MSCs were imaged at the three different time points (day 3, 7, and 10) using the ultrasound guided spectroscopic imaging. As demonstrated above, spectral analysis of the photoacoustic signals acquired at a range of wavelengths from 650 nm to 920 nm differentiated the Au NT labeled MSCs from other tissue components. Figure 6A shows 3-D combined ultrasound and spectroscopic images of the rat hind limb at different time points. The spatial distribution of the MSCs labeled with Au NTs, presented in green color, could be monitored for 10 days after the injection. Figure 6B shows quantitative analysis of the longitudinal *in vivo* imaging result. The photoacoustic signals obtained from the spatial positions of the labeled MSCs determined by the spectral analysis were summed and displayed in green color in the graph. Moreover, the photoacoustic signals from the background tissue were also analyzed and added to the graph with yellow color for the control. The Au NT concentration at day 3, 7, and 10 were able to be quantified to 2.27×10^10^ NTs/mL, 2.06×10^10^ NTs/mL, and 1.59×10^10^ NTs/mL respectively. Furthermore, this result also implies that the Au NT labeled MSCs can be detected if photoacoustic signals from the labeled cells are reasonably stronger than those from the background tissue, which are equivalent to [2.11±0.37]×10^3^ MSCs and [3.18±5.56]×10^9^ NTs/mL. Therefore, noninvasive *in vivo* longitudinal monitoring of MSCs labeled with nanotracers is capable with quantification and high sensitivity using US/PA imaging.

## Discussion

The results of this study demonstrate the feasibility of longitudinal *in vivo* monitoring of MSCs labeled with nanotracers. Once Au NT labeled MSCs are implanted into tissue, photoacoustic imaging can noninvasively detect the presence of MSCs with sufficient penetration depth (∼10–50 mm) and spatial resolution (∼20–300 µm), which are scalable depending on the specification of the transducer, such as the center frequency [21], [32]. Furthermore, Au NT labeled MSCs are able to be monitored with high sensitivity (∼10^2^–10^3^ cells) and good cell viability over a long-term period (∼2 weeks), as demonstrated with our *in vitro* and *in vivo* photoacoustic imaging results with the PEGylated fibrin gel system. As mentioned in the introduction of this paper, other stem cell imaging modalities have their own drawbacks, such as sensitivity and spatial resolution (MRI), safety and tracking period (PET and SPECT), and penetration depth (optical microscopy) [9]–[13], [31], [33]. However, US/PA imaging using Au NT labeling of MSCs can achieve various advantages simultaneously (safety, longitudinal imaging, ability to track cell distribution, high sensitivity, and high spatial resolution). Therefore, the nanotracer augmented ultrasound guided photoacoustic imaging of stem cells can be an alternative way to move beyond limitations of current stem cell imaging modalities.

In addition to monitoring the spatial distribution of the stem cells, neovascularization promoted from stem cells needs to be monitored to estimate the efficacy of the stem cell treatment. However, this work was an initial study to demonstrate the feasibility of longitudinal *in vivo* tracking using US/PA imaging. Our *in vivo* work consisted of animals which did not undergo any injury (e.g. femoral ligation), and thus neovascularization was not sufficiently promoted because the PEGylated fibrin gels with MSCs were injected into normal muscles. However, based on our previous research, we believe that the PEGylated fibrin can promote neovascularization in an ischemic muscle due to the upregulation of certain growth factors and paracrine signals as a result of the wound healing response [5]–[8]. Furthermore, using multiwavelength photoacoustic imaging, micro-blood vessels can be noninvasively imaged and quantitatively assessed without exogenous contrast agents [23], [24]. Therefore, using this technique, neovascularization developed from the implanted MSCs can be continuously monitored and distinguished from background tissue with morphological information obtained by ultrasound imaging.

US/PA imaging can be a valuable tool for tissue engineering because it allows noninvasive and longitudinal tracking of MSCs with penetration depths up to several centimeters and reasonable spatial resolution. While optical imaging is widely being used in the tissue engineering field, such as fluorescence and multiphoton imaging, it suffers from limited penetration depth (∼0.5 mm) and photobleaching [21], [32]. In contrast, the current technique provides experimentally and statistically consistent results from a minimum number of animal samples. Therefore, *in vivo* US/PA imaging can be a promising alternative for visualizing and tracking MSCs within tissue engineered constructs.

In conclusion, the presented work demonstrates the ability of the US/PA imaging to noninvasively monitor MSCs labeled with Au NTs. Au NT labeled MSCs injected intramuscularly in the lower limb of the Lewis rat were capable of being visualized using *in vivo* US/PA imaging, which indicates the ability of noninvasive monitoring of MSCs. Furthermore, the Au NT labeled MSCs were identified with high detection sensitivity *in vitro* (4.53×10^9^ NTs/mL corresponding to 1×10^4^ cells/mL and 200 cells) using ultrasound and photoacoustic imaging. In addition, Au NT labeled MSCs captured in the PEGylated fibrin gel system were able to be imaged *in vivo*, as well as *in vitro*, over a one week time period, which implies the possibility of longitudinal cell tracking using photoacoustic imaging. Accordingly, US/PA imaging using Au NT labeling of MSCs has great potential to be an alternative imaging method to monitor stem cell distribution and better understand the process of neovasularization and the wound healing response. Future work for this study includes *in vivo* monitoring of long-term MSC behaviors following an injury in order to assess and monitor the participation of MSCs in the process of neovascularization.

## Materials and Methods

### Au NT labeling of MSCs

Gold nanotracers, which are 20 nm gold nanospheres, were synthesized via citrate reduction of tetrachloroauric (III) acid (HAuCl_4_) under reflux, as described elsewhere [28]. Gold nanotracer (Au NT) cell culture medium was made by centrifuging the nanotracer solutions (5000× g for 15 minutes) and resuspending the pellet in phenol red free cell culture medium. The Au NT medium was added to the cell culture and allowed to incubate with mesenchymal stem cells (MSCs; Lonza, Walkersville, MD) for 24 hours. After 24 hours, the nanotracer medium was removed and the cells were washed with phosphate-buffered saline (PBS). Control cells were not incubated with Au NT cell culture medium. Nanotracer uptake was assessed using darkfield microscopy (Leica DMI2000B microscope equipped with a Leica DFC290 camera).

### Preparation of the PEGylated fibrin gel with Au NT labeled MSCs

The PEGylated fibrin gels were prepared by adding difunctional succinimidyl glutarate (SG)-PEG (3400 Da, 8 mg/mL in PBS without calcium; NOF America) to fibrinogen (80 mg/mL in PBS without calcium; Sigma) in a 1∶1 volume ratio. The reaction was allowed to take place at room temperature for 3–5 minute. MSCs (2×10^5^ cells/mL) were combined with the PEGylated fibrinogen mixture in a 1∶1 volume ratio. The reaction underwent gelation by adding an equal volume solution of thrombin (25 U/mL in 40 mM CaCl_2_; CalBiochem). The unbound free PEG was rinsed. The final concentrations in the gel were 10 mg/mL fibrinogen; 1 mg/mL of SG-PEG; 5×10^4^ cells/mL; and 12.5 U/mL of thrombin. The gels were then incubated at 37°C in MSC growth medium.

### Injection of the PEGylated fibrin gel with Au NT labeled MSCs

Animal handling and care followed the recommendations of the National Institutes of Health (NIH) guidelines for the care and use of laboratory animals. All protocols were approved by the Animal Care Committee of the University of Texas at Austin. Lewis rats (11 weeks old) weighing 200–250 g were used for the intramuscular injection and subsequent US/PA imaging. PEGylated fibrin injections were prepared by combining fibrinogen (40 mg/mL) and difunctional SG-PEG (4 mg/mL) in a 1∶1 volume ratio and allowing the reaction to take place at 37°C for 20 minutes. An equal volume of MSCs labeled with Au NTs were mixed with the PEGylated fibrin solution in a 1∶1 volume ratio at a concentration of 5×10^5^ cells/mL. Thrombin (25 U/mL in 40 mM CaCl_2_) was loaded into a 23 G needle. An equal volume of the PEGylated fibrin solution containing cells was then loaded into the syringe and the solution was mixed within the syringe by shaking the syringe. The final concentration of the PEGylated fibrin gel injection was 10 mg/mL fibrinogen; 1 mg/mL SG-PEG; 1×10^5^ cells/mL; and 12.5 U/mL thrombin. A total volume of 300 µL was injected in the lateral gastrocnemius of the rat. Injection depth was about 5 mm under the skin. Prior to the injection, the rat was anesthetized using 0.5–2.0% isoflurane with 1 L/min of oxygen and the hind limb of the rat was shaved.

### Tissue mimicking phantom with non-labeled and Au NT labeled MSC inclusions

The tissue mimicking phantom with non-labeled or labeled MSC inclusions was prepared for combined ultrasound and photoacoustic (US/PA) imaging. The bottom layer of the phantom was composed of gelatin (Sigma, 8% by weight) with 15 µm diameter silica particles (Sigma, 0.2% by weight) for ultrasonic scattering. The eight inclusions (20 µL each) were placed on top of the bottom layer and were composed of gelatin solutions mixed with the non-labeled or labeled MSCs suspended in cell culture medium at four different concentrations of cells (2×10^3^ cells/mL, 1×10^4^ cells/mL, 5×10^4^ cells/mL, and 2×10^5^ cells/mL). A gelatin solution with the same concentration of gelatin and silica particles as the bottom layer was used to cover the inclusions after solidification. The absorbance spectrums of all cell solutions in the eight inclusions were measured using a UV-vis spectrophotometer. The ICP-MS were used to calculate the concentrations of Au NTs for inclusions with the labeled MSCs (9.06×10^8^ Au NTs/mL, 4.53×10^9^ Au NTs/mL, 2.27×10^10^ Au NTs/mL, and 1.13×10^11^ Au NTs/mL, respectively).

### Combined ultrasound and photoacoustic (US/PA) imaging

Ultrasound and photoacoustic signals were captured using a 20 MHz array transducer with Vevo 2100 ultrasound micro imaging system (VisualSonics, Inc.) or a 25 MHz single element, focused transducer (Panametrics, Inc.) with a data acquisition board in a computer (CS8500, Gage Applied Technologies, Inc.). A tunable OPO laser beam (Premiscan, GWU, Inc.) pumped by a pulsed Nd:YAG laser (Quanta-Ray, Spectra Physics, Inc.) was delivered through an optical fiber bundle (Ceramoptec, Inc.). The fiber bundle was combined with the ultrasound transducer and aligned such that the transmitted light would focus at the focal point of the transducer. The trigger signal from the laser system was synchronized with the computer or the Vevo 2100 imaging system to capture the photoacoustic signal when the pulsed laser irradiated. Once the photoacoustic transient and ultrasound pulse-echo signal was captured, the off-line image processing was performed with the acquired imaging data. The ultrasound and photoacoustic signals were bandpass filtered (5–45 MHz) to reduce noise. The magnitudes of the ultrasound and photoacoustic signals were calculated by taking the absolute values of analytic signals obtained using the Hilbert transform. The photoacoustic signal magnitude was then compensated for wavelength dependent laser fluence. The compensated ultrasound and photoacoustic signals were interpolated to 2D images. The combined ultrasound and photoacoustic images were created by overlaying photoacoustic intensities higher than a user-defined threshold on the grayscale ultrasound images.

## Supporting Information

Figure S1 **Optical selectivity of photoacoustic imaging for spectral analysis.** (A) The normalized absorbance spectra of Au NTs (top) and the Au NT labeled MSCs (middle). The green shaded region shows the optical window in biological tissue, which is the optimal wavelength ranges for *in vivo* imaging. Comparison within the optical window of the normalized absorbance spectra of labeled MSCs, oxygenated and deoxygenated hemoglobin (bottom). (B) Photoacoustic signal amplitude from the inclusions with 5,000 MSCs at multiple wavelengths (532, 600, 650, 700, 750, and 800 nm).
(TIFF)

Figure S2 **Cell viability after laser irradiation.** Diagram of the experimental setup for laser irradiation (left) and fluorescent images of LIVE/DEAD stained MSCs loaded with nanotracers (right). MSCs were irradiated with 50 laser pulses at 532 nm wavelength. Cells remain viable after irradiation at laser fluencies reaching 17 mJ/cm^2^, but cell death was observed for laser fluence higher than 26 mJ/cm^2^.
(TIFF)

## References

[1] Lloyd J (2010). Heart Disease and Stroke Statistics-2010 Update: A Report From the American Heart Association (vol 121, pg e46, 2010).. Circulation.

[2] Liu J, Hu Q, Wang Z, Xu C, Wang X (2004). Autologous stem cell transplantation for myocardial repair.. Am J Physiol Heart Circ Physiol.

[3] Boyle AJ, Schulman SP, Hare JM (2006). Is stem cell therapy ready for patients? Stem cell therapy for cardiac repair - Ready for the next step.. Circulation.

[4] Segers VFM, Lee RT (2008). Stem-cell therapy for cardiac disease.. Nature.

[5] Zhang G, Wang XH, Wang ZL, Zhang JY, Suggs L (2006). A PEGylated fibrin patch for mesenchymal stem cell delivery.. Tissue Engineering.

[6] Zhang G, Suggs LJ (2007). Matrices and scaffolds for drug delivery in vascular tissue engineering.. Advanced Drug Delivery Reviews.

[7] Zhang G, Hu QS, Braunlin EA, Suggs LJ, Zhang JY (2008). Enhancing efficacy of stem cell transplantation to the heart with a PEGylated fibrin biomatrix.. Tissue Engineering Part A.

[8] Zhang G, Drinnan CT, Geuss LR, Suggs LJ (2010). Vascular differentiation of bone marrow stem cells is directed by a tunable three-dimensional matrix.. Acta Biomaterialia.

[9] Zhou R, Acton PD, Ferrari VA (2006). Imaging stem cells implanted in infarcted myocardium.. Journal of the American College of Cardiology.

[10] Wu JC (2007). Comparison of imaging techniques for tracking cardiac stem cell therapy.. Journal of Nuclear Medicine.

[11] Beeres S, Bengel FM, Bartunek J, Atsma DE, Hill JM (2007). Role of imaging in cardiac stem cell therapy.. Journal of the American College of Cardiology.

[12] Sheikh AY, Wu JC (2006). Molecular imaging of cardiac stem cell transplantation.. Curr Cardiol Rep.

[13] Schroeder T (2008). Imaging stem-cell-driven regeneration in mammals.. Nature.

[14] Wang B, Yantsen E, Larson T, Karpiouk AB, Sethuraman S (2009). Plasmonic Intravascular Photoacoustic Imaging for Detection of Macrophages in Atherosclerotic Plaques.. Nano Letters.

[15] Mallidi S, Larson T, Tam J, Joshi PP, Karpiouk A (2009). Multiwavelength Photoacoustic Imaging and Plasmon Resonance Coupling of Gold Nanoparticles for Selective Detection of Cancer.. Nano Letters.

[16] Park S, Karpiouk AB, Aglyamov SR, Emelianov SY (2008). Adaptive beamforming for photoacoustic imaging.. Optics Letters.

[17] Yoon SJ, Mallidi S, Tam JM, Tam JO, Murthy A (2010). Utility of biodegradable plasmonic nanoclusters in photoacoustic imaging.. Optics Letters.

[18] Su JLS, Wang B, Emelianov SY (2009). Photoacoustic imaging of coronary artery stents.. Optics Express.

[19] Oraevsky A, Karabutov A, Vo-Dinh T (2003). Optoacoustic Tomography.. Biomedical Photonics Handbook.

[20] Xu MH, Wang LHV (2006). Photoacoustic imaging in biomedicine.. Review of Scientific Instruments.

[21] Emelianov SY, Li PC, O'Donnell M (2009). Photoacoustics for molecular imaging and therapy.. Physics Today.

[22] Emelianov SY, Aglyamov SR, Karpiouk AB, Mallidi S, Park S (2006). Synergy and Applications of Combined Ultrasound, Elasticity, and Photoacoustic Imaging..

[23] Hu S, Maslov K, Wang LHV (2009). Noninvasive label-free imaging of microhemodynamics by optical-resolution photoacoustic microscopy.. Optics Express.

[24] Zhang HF, Maslov K, Stoica G, Wang LV (2006). Functional photoacoustic microscopy for high-resolution and noninvasive in vivo imaging.. Nat Biotechnol.

[25] Daniel MC, Astruc D (2004). Gold nanoparticles: Assembly, supramolecular chemistry, quantum-size-related properties, and applications toward biology, catalysis, and nanotechnology.. Chemical Reviews.

[26] Boisselier E, Astruc D (2009). Gold nanoparticles in nanomedicine: preparations, imaging, diagnostics, therapies and toxicity.. Chemical Society Reviews.

[27] Jain PK, Lee KS, El-Sayed IH, El-Sayed MA (2006). Calculated absorption and scattering properties of gold nanoparticles of different size, shape, and composition: Applications in biological imaging and biomedicine.. Journal of Physical Chemistry B.

[28] Ricles LM, Nam SY, Sokolov K, Emelianov SY, Suggs LJ (2011). Function of mesenchymal stem cells following loading of gold nanotracers.. International Journal of Nanomedicine.

[29] Mallidi S, Larson T, Aaron J, Sokolov K, Emelianov S (2007). Molecular specific optoacoustic imaging with plasmonic nanoparticles.. Optics Express.

[30] (2011).

[31] Li SC, Tachiki LML, Luo J, Dethlefs BA, Chen ZP (2010). A Biological Global Positioning System: Considerations for Tracking Stem Cell Behaviors in the Whole Body.. Stem Cell Reviews and Reports.

[32] Wang LV (2008). Prospects of photoacoustic tomography.. Medical Physics.

[33] Kraitchman DL, Heldman AW, Atalar E, Amado LC, Martin BJ (2003). In vivo magnetic resonance imaging of mesenchymal stem cells in myocardial infarction.. Circulation.

