# Supplementary figures and images for: In vivo Ultrasound and Photoacoustic Monitoring of Mesenchymal Stem Cells Labeled with Gold Nanotracers
Source: PLoS One. 2012 May 16;7(5):e37267. doi: 10.1371/journal.pone.0037267 (PMC3353925; doi:10.1371/journal.pone.0037267)

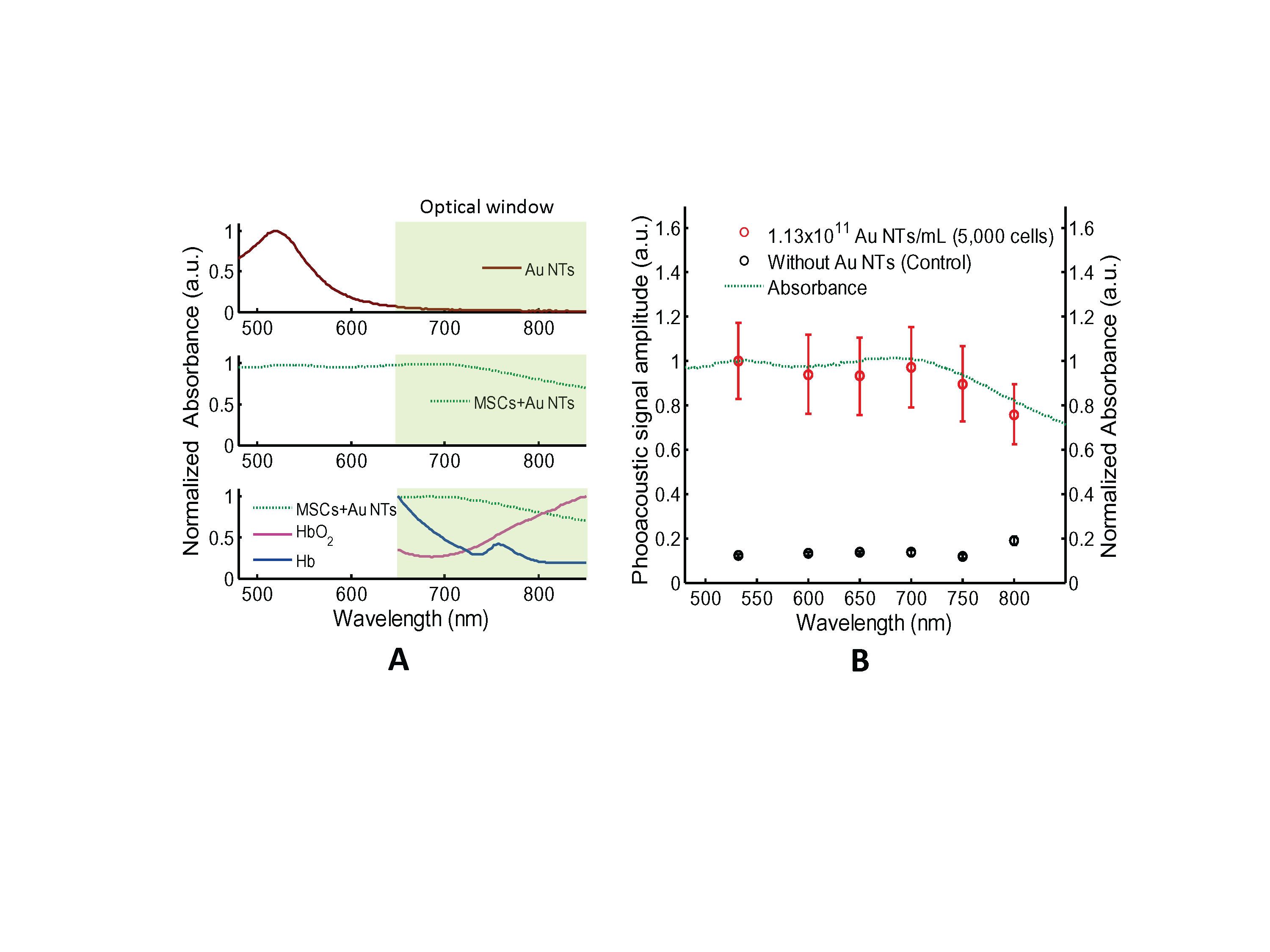

Supplement: Figure S1 — Optical selectivity of photoacoustic imaging for spectral analysis. (A) The normalized absorbance spectra of Au NTs (top) and the Au NT labeled MSCs (middle). The green shaded region shows the optical window in biological tissue, which is the optimal wavelength ranges for in vivo imaging. Comparison within the optical window of the normalized absorbance spectra of labeled MSCs, oxygenated and deoxygenated hemoglobin (bottom). (B) Photoacoustic signal amplitude from the inclusions with 5,000 MSCs at multiple wavelengths (532, 600, 650, 700, 750, and 800 nm). (TIFF) [file pone.0037267.s001.tiff]

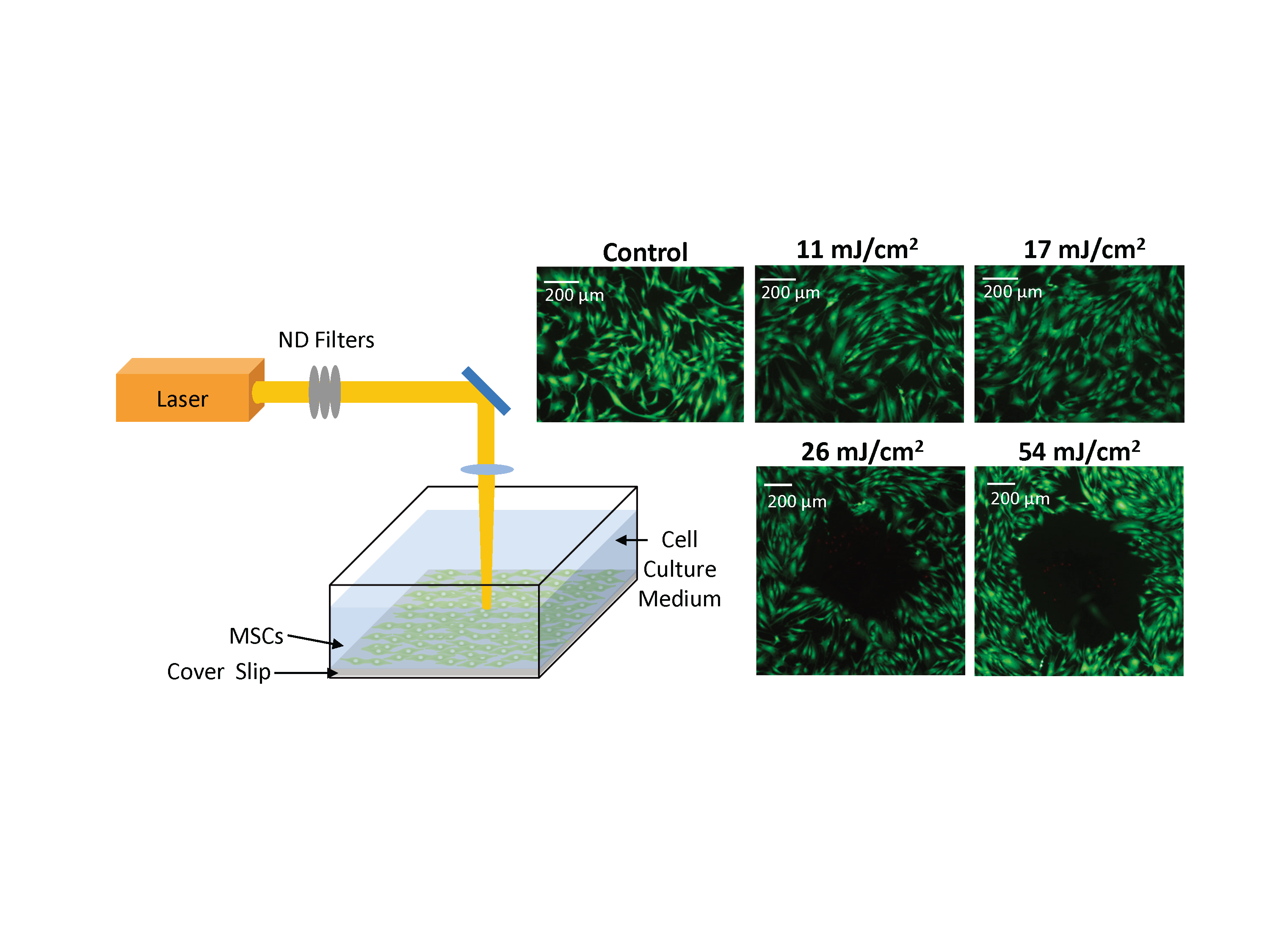

Supplement: Figure S2 — Cell viability after laser irradiation. Diagram of the experimental setup for laser irradiation (left) and fluorescent images of LIVE/DEAD stained MSCs loaded with nanotracers (right). MSCs were irradiated with 50 laser pulses at 532 nm wavelength. Cells remain viable after irradiation at laser fluencies reaching 17 mJ/cm2, but cell death was observed for laser fluence higher than 26 mJ/cm2. (TIFF) [file pone.0037267.s002.tiff]
